# Nesting Environment Provides Sex-Specific Neuroprotection in a Rat Model of Neonatal Hypoxic-Ischemic Injury

**DOI:** 10.3389/fnbeh.2018.00221

**Published:** 2018-10-02

**Authors:** Briana Mason, L. G. Rollins, Evans Asumadu, Christina Cange, Najah Walton, S. Tiffany Donaldson

**Affiliations:** ^1^Developmental and Brain Sciences, Department of Psychology, University of Massachusetts Boston, Boston, MA, United States; ^2^Clinical Psychology Program, Department of Psychology, University of Massachusetts Boston, Boston, MA, United States; ^3^Warren Alpert Medical School, Department of Psychiatry, Brown University, Providence, RI, United States

**Keywords:** Rice-Vanucci P7 HI model, Long Evans rats, environmental enrichment, Morris water maze, neurogenesis, neonatal hypoxic ischemic injury, hippocampus

## Abstract

Hypoxic-ischemic (HI) encephalopathy is a devastating injury that occurs when the fetal brain is deprived of oxygen and blood to a degree that may lead to neurological damage, seizing and cerebral palsy. In rodents, early environmental enrichment that promotes maternal care-taking behavior (mCTB) can improve neurobehavioral outcomes and protect against neurological decline. We hypothesized that an enhanced nesting environment would improve mCTB as measured by pup weight gain, and support greater HI recovery in developing rats. Pregnant dams (E15-16) were introduced to either control Standard Facility (SF) housing or closed nestbox (CN) conditions and maintained in larger cages through pup weaning. On postnatal day (PND) 7, male and female Long-Evans rat pups (*N* = 73) were randomly sorted into one of two surgical conditions: control and HI. HI pups received isoflurane anesthesia and right carotid artery ligation, a 2-h rest followed by 90 min exposure to a moist hypoxic (92% N, 8% O2) chamber. Pups (PND 8) were weighed daily, and tested on the Morris Water Maze (MWM) task (PND 35-50). Results demonstrate significant differences afforded to male and female pups based on weight measure, where CN-rearing modifies pre-weaning adolescent weights in females and increases post-weaning weights in males and females by an average of 10 g. Following successful MWM training and acquisition (PND 35-37), both male and female CN-raised animals demonstrated faster latency to find the hidden platform (HP) during HP trials (PND 38-42) and appeared to freely explore the MWM pool during an additional probe trial (PND 43). Moreover, after sacrifice (PND 60), CN rearing created sex-specific alterations in brain-derived neurotrophic factor (BDNF), glial-derived neurotrophic factor (GDNF) immunopositive cell staining of the dorsomedial striatum and CA1 of the hippocampus. CN-rearing afforded HI males higher BDNF levels in the striatum and produced greater GDNF levels in the hippocampus of HI-injured females. These results suggest that early life environmental enrichment positively modifies nesting environment, increases weight gain, as well as spatial learning and memory in a sex-specific directionality. Our findings also implicate correlative changes in corticolimbic neurotrophin protein levels in the CN-reared animals that may contribute to these benefits.

## Introduction

Neonatal Hypoxic-ischemic encephalopathy (HIE) is a serious neurological injury resulting from oxygen and glucose deprivation during birth. Current estimates predict that 1–2 of every 1,000 full term neonates develop HIE (Volpe, 2009). Surviving infants may go on to develop long-term disabilities, including reoccurring seizures (Silverstein and Jensen, 2007; Björkman et al., 2010), motor impairments (Martinez-Biarge et al., 2011) and attentional and learning deficits (Lou, 1996; Perez et al., 2013).

Cognitive deficits are a hallmark of neonatal HIE, with some studies estimating 70% of adolescent HIE survivors demonstrate some degree of cognitive difficulty and 50% require special educational services (Lindström et al., 2006). Using the Rice-Vannucci method (Vannucci et al., 1999), the most prevalent rodent model of neonatal HIE, the injury is modeled in rats at postnatal day (PND) 7 by ligation of the right common carotid artery followed by oxygen deprivation. This model simulates both morphometric and functional sequalae of term neonatal hypoxic ischemic (HI) injury, including spatial and working memory deficits (Smith et al., 1991; Balduini et al., 2000; de Paula et al., 2009). The bilateral hippocampus and frontocorticolimbic system are highly vulnerable to HI injury, with studies suggesting that these areas are damaged in an estimated 90% of HI models and infarcted in another 56% of subjects (Rice et al., 1981). As a result of HI, early cell death occurs in the neocortex, and a second wave of cell death occurs from 12 h to 24 h after oxygen deprivation, targeting subcortical structures including the hippocampus, striatum and thalamus (Azzarelli et al., 1996; Northington et al., 2007).

Early environmental enrichment with large cages, social peers and sensorimotor stimulation has been shown to somewhat improve the functional memory outcomes of adolescent but not adult female rats with neonatal HI injury, without modifying the hippocampal or striatal damage (Pereira et al., 2008). Maternal care-taking behavior (mCTB) provided by rat dams in the first 2 weeks of life has also been shown to alter the stress response of offspring through direct modifications of the hypothalamic-pituitary-adrenal axis (Meaney, 2001) and neurotrophin levels in the central amygdala (Berman et al., 2014). During the pre-weaning period, greater licking and grooming, and arched back nursing result in the regulation of emotional as well as neural systems (Caldji et al., 1998; Roth and Sweatt, 2011). Pre-weaning environmental enrichment has been successfully applied to promote typical development in animal models (Koo et al., 2003; Szabadfi et al., 2009) and to improve learning and memory through alterations in hippocampal proteins and synapse formation (Venable et al., 1989; Bredy et al., 2003).

Early handling (Chou et al., 2001) and environmental enrichment during early life (Pereira et al., 2008) are neuroprotective in HI injured animals working via morphological changes to dendritic spine density and synaptic branching (Rojas et al., 2013; Zhao et al., 2013). In addition, this neuroprotection involves changes in the expression of neurotrophins like brain-derived neurotrophic factor (BDNF) and glial-derived neurotrophic factor (GDNF; Lin et al., 1993; Skaper, 2012). Treatment with exogenous BDNF reduces HI-induced spatial deficits in rats trained on a Morris Water Maze (MWM) task (Almli et al., 2000). GDNF is a neurotrophin present in dopaminergic neurons that has been characterized as a marker of neuronal survival (Wang et al., 2004; Bakshi et al., 2006). Collectively, BDNF and GDNF are thought to act as endogenous neuroprotective agents (Kiprianova et al., 1999; Allen et al., 2013; Chen et al., 2013). The outcome of children who have experienced a brain injury has been correlated to levels of these two proteins, such that decreases in BDNF and GDNF indicate greater working memory deficits (Chiaretti et al., 2003). These findings indicate that environmental interventions may be a potential avenue for prevention or treatment of the dramatic cognitive deficits that often follow HI. Environmental enrichment has been shown to benefit individuals suffering with neurodegenerative diseases such as Huntington’s, Parkinson’s, or Alzheimer’s diseases, through the utilization of the brain’s plastic nature (for review, see Laviola et al., 2008). Although environmental enrichment has been well studied in terms of neural plasticity and recovery in many models of neural injury and disease there are few studies that investigate the effects of the early environment on developmental trajectory after HI, and fewer still that seek to understand the interplay between the quality of early environment and cognitive sequelae.

This study was designed to address the complexity of functional outcomes, while simultaneously examining potential proteomic changes as they reflect differences in recovery. We introduced rat dams to a closed nestbox (CN) condition as a protected environment as compared to the standard facility (SF) conditions, hypothesizing that the sheltered environment would provide positive enrichment for dams and pups during the pre-weaning period (Mychasiuk et al., 2012). We further hypothesized that the CN environment would improve phenotypic and proteomic features of HI in male and female pups in a sex-dependent manner. This potential benefit was theorized based on several sex-specific effects observed in environmental enrichment, pup weight gain and HI injury. The pattern of damage following HIE is thought to be different between males and females, with females displaying significantly less injury than males in physical and cognitive domains (Zhu et al., 2006; Hill and Fitch, 2012; Smith et al., 2014). Sex differences may also determine the effectiveness of treatment for HIE, to which end pre-weaning environmental modification may be useful (Fan et al., 2011; Nie et al., 2016).

## Materials and Methods

### Animals

**Figure 1:**
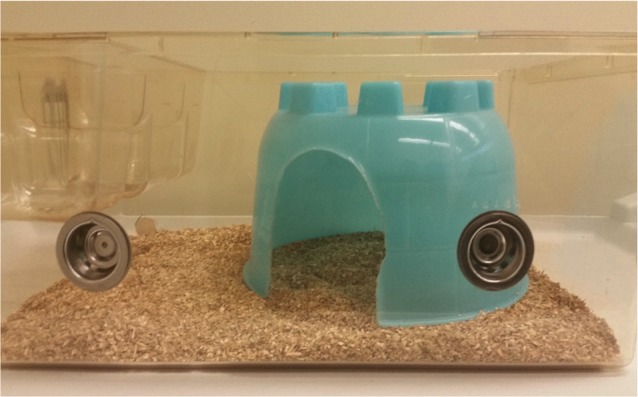
Picture of Closed Nestbox (CN) inside standard cage with bedding. CN was added to the dam’s cage at embryonic day (ED) 10 (E10-15) and remained until weaning on postnatal day (PND) 21.

**Figure 2:**
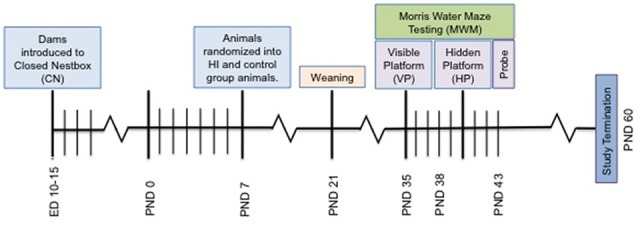
Depiction of study timeline indicating the introduction of the CN enrichment at embryonic day 10–15, surgery on PND 7, Morris Water Maze (MWM) testing and study termination.

All procedures utilized in this experiment were approved by the University of Massachusetts Boston Institutional Animal Care and Use Committee and closely followed applicable portions of the Animal Welfare Act and the U.S. Department of Health and Human Services’ “Guide for the Care and Use of Laboratory Animals.” Pregnant Long Evans rats (*N* = 7; embryonic day 10) were purchased from Charles River (Wilmington, VA, USA) and upon arrival, were singly housed and randomly assigned to SF or CN (Figure 1) conditions in the animal vivarium in a light- and temperature-controlled environment at 22°C with lights on at 07:00 h and off at 19:00 h. Dams were kept in a Plexiglas cage with dimensions 31.75 cm × 41.7 cm × 17.8 cm. CN conditions were similar, but also included a small (7.75″L × 6″W × 4.5″H), opaque plastic shelter that contained one entrance placed in the center of the Plexiglas cage. Plastic shelters were inserted in the nesting environment of pregnant dams on embryonic day 10 and left undisturbed until time of delivery. After the dams gave birth, litters were sexed and humanely culled to 10–12 pups with even distribution of males and females in order to limit dramatic variations in maternal care (Champagne et al., 2003). Litters were also relatively stable in male to female ratio, never exceeding more than 1:3 females to males. Individual pups within litters were excluded from the research paradigm if they failed to reach a weight standard of 11 g at PND7 during weight check-in during randomization. Ten litters with a total of 73 pups were randomized within litters into one of two surgical conditions: control (*n* = 17 females, *n* = 18 males) and hypoxia ischemia (*n* = 19 females, *n* = 19 males; Table 1). The study timeline is depicted in Figure 2.

**Table 1:** Total number of subjects, separated by sex and housing condition.

Subject Grouping	Standard Facility		Closed Nestbox (CN)	
	Male	Female	Male	Female
Control	*n* = 11	*n* = 11	*n* = 7	*n* = 6
Hypoxia-Ischemia	*n* = 7	*n* = 9	*n* = 12	*n =* 10
Total	18	20	19	16

### Surgery

On PND 7, male and female pups in each housing condition were randomly sorted within litters into one of two surgical conditions: HI and control groups. Animals in the HI condition underwent carotid artery ligation and hypoxia according to the Levine model of HIE, using the Rice-Vannucci modification (Rice et al., 1981). Pups were anesthetized with 3%–5% isofluorane and maintained on 1%–2% for the duration of the surgical procedure. We performed double ligation and severed the right common carotid artery followed by a 2 h rest period with the dam. HI pups were then removed from the litter and placed into a plastic container and supplied exclusively with 8% Oxygen and 92% Nitrogen for 90 min and then returned to their dams. Within this chamber, temperature was kept at a steady 36°C to mimic normative nesting conditions (Mortola and Dotta, 1992; Cameron et al., 2000). Animals in the control group remained in their home cage and were only removed from their dams to be marked for identification. In the current study, the mortality rate following right carotid artery ligation and post-operative 90-min hypoxia was 5% (two out of 40 total animals; one male and one female). This mortality rate is well within reported standards following HI induction in animals (Nakajima et al., 2000; Demers et al., 2005; Pereira et al., 2008). On PND 21, animals were weaned and housed by sex with littermates, with 2–3 animals per cage.

### Body Weight

Animals were weighed daily (grams) from PND 8–21 and every 3–4 days from PND 22–60. From PND 8–14, animals were weighed on a small plastic Sterilin weighboat (ThermoFischer, Cambridge, MA, USA), as appropriate for their size and physiological condition. Upon their maturation as denoted by the full appearance of fur at PND 15, animals were weighed in a closed-lid metal weight cage zeroed on an electronic scale (Basic Electronic Scale, Mettler Toledo International, Inc., Billerica, MA, USA).

### Morris Water Maze—Visible Platform

All testing for the MWM trials took place within a pool (120.65 cm in diameter, 176.25 cm in height at its deepest point) filled with water heated to room temperature (±25–27°C) and colored with non-toxic milk powder. Around the pool, key shapes were pasted for internal orientation to the surrounding directions of North (N), West (W), East (E) and South (S) as reference cues for the rats.

PND 35 was the first day of testing, selected based on previous research suggesting that rats are able to perform with mature cognition by PND 30 (Ikeda et al., 2001; Arteni et al., 2003). On the initial test day, animals were placed on the fixed, visible platform (VP) that was marked by bright green tape in the SE corner of the pool for 15 s, and were allowed to swim freely for up to 20 s Rats were tested in four trials each day from PND 35–42 and were given up to 60 s to find the VP on each trial. If rats did not find the platform within 60 s they were guided to the platform and were left on the platform for 10 s to familiarize them with its location. Each rat was dried and warmed between each trial and given a 10 min inter-trial interval throughout the four-trial testing period. The latency to reach the platform was recorded from trial to trial. Tests were conducted in a low light setting, with the experimenter standing at a distance out of view of the animals. Ethovision XT11 software and a digital camera (Microsoft Webcam, 12.0; Microsoft, Redmond, Washington, USA) were used to video record information for 78 of the 104 subjects.

For VP training, rats were tested from PND 35–38, and were dropped off from the South, North, East and West for each of the 3 days. The platform remained fixed in the SE corner. For the hidden platform (HP) trials, the platform was made translucent and remained fixed in the SW corner of the pool 0.5 inches under the surface of the opaque water. Each day, animals were placed in the pool in the directions of N, N, NW and NW for a total of four trials. Animals were tested in HP from PND38–42.

After completing VP and HP testing, we conducted probe trials (PND43) and captured video information for these trials with a subset of animals. Probe trials consisted of a 60 s swimming period for each animal, without a platform. For 60 s animals were allowed to freely swim around the pool and swimming behavior was coded for proximity to the most recent location of the platform.

### Brain Morphology and Immunofluorescence

Animals were sacrificed at PND60 with the aid of a restraint cone (Harvard Apparatus, Holliston, MA, USA) and live decapitation. Brains were extracted and snap frozen with 2-methylbutane chilled on dry ice, and then stored at −80°C until the time of post-fixation and cryoprotection (increasing 5%–20% sucrose + 4% paraformaldehyde).

Following this, brain tissues were microsectioned at 20–25 μm (Leica CM 3050S; Leica Biosystems, Buffalo Grove, IL, USA) taken at Bregma 4.70–5.60 mm for the striatum (Paxinos and Watson, 2004) and Bregma −3.80 to −04.16 mm for the hippocampus thaw-mounted and placed directly on adhesive slides (SuperFrost Plus; ThermFischer, Cambridge, MA, USA). Mircosections were randomly divided into Nissl, control and positive immunofluorescent groups to include 5–6 animals per treatment and housing group. For Nissl stains, first we removed the fat (95% EtOH, 15 min) then rehydrated (70% and 50% EtOH, 5 min each). Next we ran the sections through dH_2_O washes (1–2 min) followed by immersion in Cresyl Violet (Sigma, 0.1% (2–4 min); this was again followed by dH_2_O wash (1 min). After, we returned to dehydration steps (50% EtOH (1 min), 75% acid EtOH, 95% and 100% EtOH (2 min) and the clearing agent, Histoclear (National Diagnostics, Atlanta, GA, USA). In models of HI injury, necrosis and apoptosis (Northington et al., 2007) are assessed close in time to the injury (see also Cai et al., 2008). Given that we sacrificed the animals ~PND60, nearly 2 months after the HI injury, we instead think that information about the size of the infarct and relative damage to the hippocampus is important for the data presented in this manuscript (Ikeda et al., 2001).

After, sections were fixed with 4% paraformaldehyde for 30 min at a time and, repeatedly rinsed with phosphate buffered saline (PBS) for 10 min per wash. After five washes, slides were soaked in bovine serum albumin for 30 min, and then were covered with the primary antibodies BDNF (1:1,000; ThermoFischer, Cambridge, MA, USA) and GDNF (1:500, ThermoFischer, Cambridge, MA, USA) overnight. The next day, slides were recovered from the humidity chamber and washed with PBS again for 10 min per wash. Following this, slides were coated with two fluorophore conjugated secondary antibodies for approximately 1 h: goat anti-rabbit AlexaFluor 488 (IgG H&L) and goat anti-mouse AlexaFluor 647 (IgG H&L) at a concentration of 1:600 (Abcam, Cambridge, MA, USA and ThermoFischer, Cambridge, MA, USA). Excess antibody was washed with PBS three times, with 10 min per wash. Fluorescence was enhanced with anti-fade reagent (Prolong anti-fade reagent with 6-diamidino-2-phenylindole (DAPI); ThermoFischer, Cambridge, MA, USA), and slides were coverslipped. To preserve apparent fluorophores, slides were coated with clear nail polish around the edges to prevent oxidation. Slides were imaged within the week, with up to six images taken for each bilateral brain area of interest. Fluorescent images were converted from native high resolution .CZI to .JPEG format and dropped in ImageJ (National Institutes of Health, online). A 500 × 500 pixel yellow-colored region of interest (ROI; 2.23 μm per pixel) was manually drawn on the center of each image, and positional consistency was maintained through the use of an ImageJ placement macro (six images per subject, two merges in total overlap; 12 images if images were found to be ambiguous).

ImageJ was used to set color thresholds to maintain consistency for each fluorophore type and were subsequently processed using ImageJ to avoid experimenter bias and to increase objectivity. Initial GDNF counts proved inconsistent and variable across groups, measurement was altered to suit the diversity of the stain-type. Measurement analysis of GDNF fluorescent intensity level was restricted to a 500 × 500 pixel (2.23 μm per pixel) square. Circularity and overall size of GDNF-tagged cells were relatively unrestricted, as the main factor analyzed was mean brightness of areas contained within centered ROIs.

### Data Analysis

All data were coded and interpreted with the use of SPSS 22.0 and 23.0 (Windows 10 and Mac 6.1 compatible versions). Body weight was analyzed using a Generalized Linear Mixed Model (GLMM) with repeated measures, with Housing and Surgical conditions. Each analysis was done separately for males and females. MWM trials were analyzed using GLMM with repeated measures. The latency to reach the platform was assessed according to Housing Condition (SF or CN) and Surgical Condition (Control or HI) across the four trials of each testing day. Each analysis was done separately for males and females.

For the probe trial, collected data were analyzed with the use of the Ethovision XT11 (Leesburg, VA, United States). The MWM area was divided into four quadrants based on location of the symbols placed around the pool (NW, NE, SW, SE) and time spent in each quadrant was analyzed by the tracking system in sec. Videos were randomly coded for each subject and then run through data analysis for the variables of Movement (gauging the swimming style of the animal from its center) and Speed (how quickly an animal moved throughout the pool) as controls based on possible physical deficits between groups (Bona et al., 1997). Swimming speed was determined as also given by the independent variables of Housing Condition, Surgical Condition. The significance was set at probability of 0.05 or less. Confidence intervals were set at 95%.

A two-way between-subjects ANOVA was performed for the factors of Surgical condition (Control or Hypoxia-Ischemia) and housing condition (SF or CN) to compare means in BDNF and DAPI levels, as well as modifications to average GDNF levels based on fluorescent intensity following fluorescent microscopy in the dorsomedial striatum and CA1 of the hippocampus of subjects. All data was tested for normality. For two of the dependent measures, striatal counts of BDNF in males and striatal DAPI levels in females, homogeneity of collected data was not met under Levine’s Test. While this was not complete unexpected due to the possible variability in targeted tissue, data were adjusted to reflect normal distributions through the exclusion of BDNF striatal counts in two male subjects and through the exclusion of DAPI striatal counts in one female subject.

## Results

### Brain Morphology

Due to the severity of the injury sustained in the SH group, there was not enough intact tissue from this group to yield data from measurements of cortical or hippocampal area that could be statistically compared in a meaningful way. However, the lack of tissue is in itself an important finding that distinguishes animals from the two housing conditions. Therefore, the results from the measurements of hippocampal area will be reported in descriptive terms.

**Figure 3:**
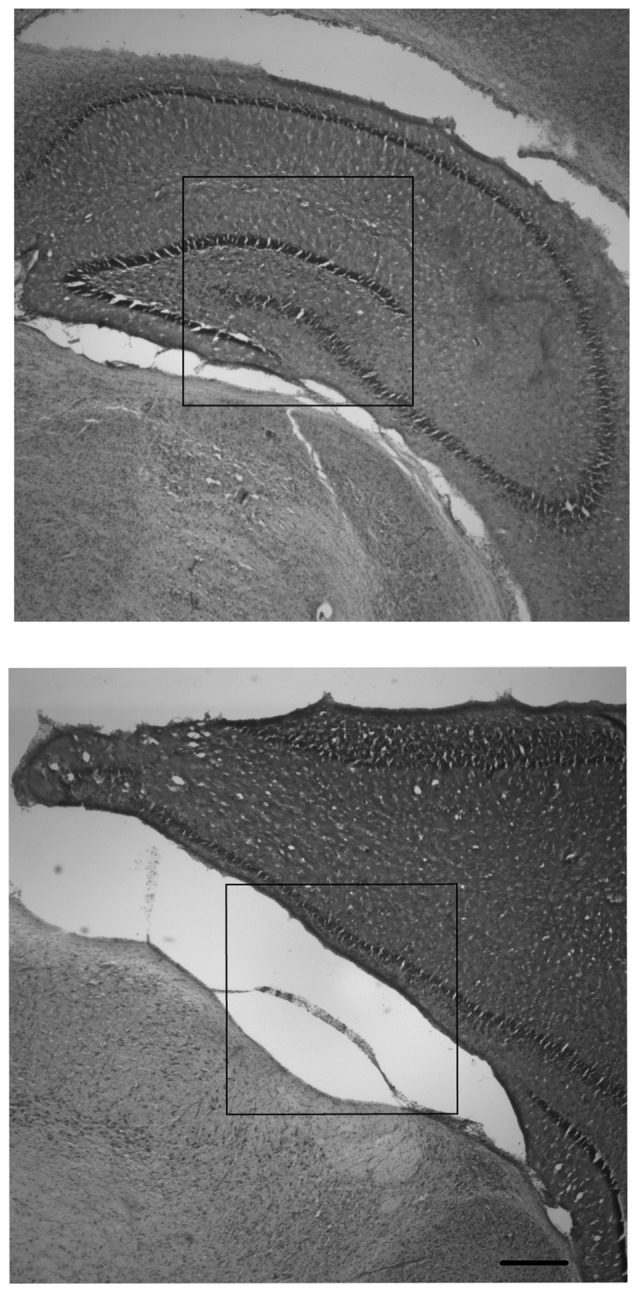
Nissl-stained coronal hippocampal sections (ipsilateral to injury) from representative males exposed to Hypoxic-ischemic (HI) injury and reared in CN (top panel) and Standard Facility (SF; bottom). CN rearing provided enduring neuroprotection in late adolescence following neonatal HI insult. Scale bar = 200 μm for all images (bottom right); 20× magnification.

Observations of right and left hippocampal areas revealed that all CN animals retained tissue in both right and left hippocampal areas, whereas there were no SF animals which had a visible right hippocampus (ipsilateral to the injury). For SF animals, there were either large infarcts where the right hippocampus would be in un-injured animals, or the hippocampal area was not present in tissue that was intact. Females in the SF group had some visible left hippocampus tissue (contralateral to the injury), whereas SF males did not have any visible hippocampal tissue in the left hemisphere. In CN animals, all animals had both right and left hippocampi, although each displayed a disparity between right and left hippocampi, with the right measuring smaller than the left. Measurements of the cortical area of the right and left hemispheres showed some asymmetry between hemispheres, with the right (ipsilateral) hemisphere measuring smaller than the left (contralateral) hemisphere as expected. However, the degree of disparity appeared to differ greatly between groups. There was a dramatic disparity between right and left hemispheres for both males and females in the SF group, but comparatively little disparity in CN animals (Figure 3). This indicates that on average, regardless of sex, CN animals had less damage from infarct or atrophy in the ipsilateral hemisphere than SF animals.

### Body Weight

Pre-weaning weight measurements were collected daily from PND 8 to PND 21 and adolescent weights were collected every 3–4 days from PND 24–49. There were no significant differences between surgical groups or housing conditions for males during this pre-weaning period. However, pre-weaning female weights were significantly different between surgical conditions, with HI animals weighing significantly less than control animals (*F*_(1,34.02)_ = 5.80, *p* < 0.05). Analysis of adolescent weights yielded significant differences between housing conditions for males and females in the HI condition (*F*_(1,32.00)_ = 6.25, *p* < 0.01), with CN animals weighing an average of 10 grams more than SF animals. There were no other significant differences between conditions.

### Morris Water Maze Performance

#### Visible Platform Training

**Figure 4:**
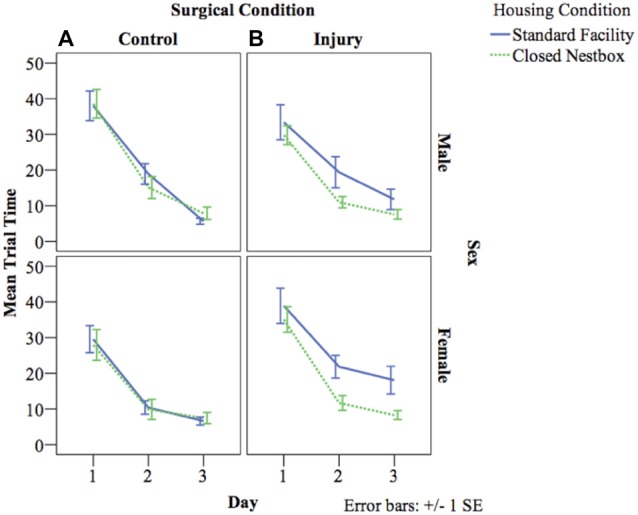
**(A,B)** Mean latency by study condition in visible platform (VP) training. The following graphs mark the average latency of animals to reach the platform over the course of the four water maze trials each day for three consecutive days. The lines compare average latency for animals in the CN and SF housing conditions according to surgical condition: **(A)** Control, **(B)** HI Injury. Only main effects were observed for latency for males (*F*_(2)_ = 103.20, p < 0.05), and females (*F*_(2)_ = 71.30, p < 0.05) with both demonstrating decreased latency across the three training days indicating successful task acquisition.

VP training allows animals to learn the water maze testing procedure, and also measures spatial memory acquisition. The latency for animals to reach the platform decreased over the 3 days of VP testing indicating successful memory acquisition across study conditions for males (*F*_(2,462)_ = 103.20, *p* < 0.05), and females (*F*_(2,3478)_ = 71.30, *p* < 0.05). For females, there were significant differences between surgical conditions in latency to reach the platform regardless of housing condition (*F*_(1,29)_ = 8.02, *p* < 0.05), with control animals performing significantly better than HI animals. Unexpectedly, there was no significant effect of housing condition for males or females on latency to reach the platform independent of other study factors, and there were no significant interaction effects between study variables (Figure 4).

#### Hidden Platform Testing

**Figure 5:**
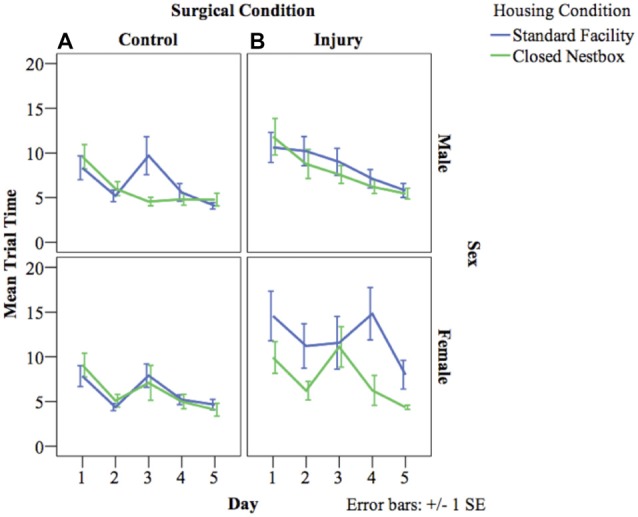
**(A,B)** Mean latency by surgical and housing condition in hidden platform (HP) testing for males and females. The following graphs mark the average latency of animals to reach the HP over the course of the four trials each day for five consecutive days. The lines compare average latency for animals in the CN and SF housing conditions according to surgical condition: **(A)** Control, **(B)** HI Injury. There were significant differences between surgical conditions regardless of housing condition for males (*F*_(1)_ = 11.35 *p* < 0.001; HI males relative to Control males). Females demonstrated a significant interaction effect between surgical condition and housing condition (*F*_(1)_ = 9.37 *p* < 0.001; HI SF as compared to all other groups, same sex), with HI SF females performing significantly worse than all other groups.

HP testing with a stationary platform location, requires animals to develop memory strategies using spatial cues without the visual cue of the platform. A decrease in latency across days indicates functional long-term memory. There was a significant decrease in latency by day indicating functional long-term memory across treatment and surgical conditions for males (*F*_(4,800)_ = 8.57, *p* < 0.05), and females (*F*_(4,522)_ = 6.08, *p* < 0.05). For males, there was a significant effect of surgical condition on time to locate the platform, with HI animals performing significantly worse than control animals (*F*_(1,800)_ = 11.35 *p* < 0.001). There were no significant differences for males according to housing condition, or significant interaction effects between study factors. Females demonstrated a significant interaction effect between surgical condition and housing condition (*F*_(1,522)_ = 9.37 *p* < 0.001), with HI SF females performing significantly worse (12.04 s compared to HI CN (7.58 s), Control CN (6.06 s) and Control SF (6.00 s; Figure 5).

#### Probe Trial

**Figure 6:**
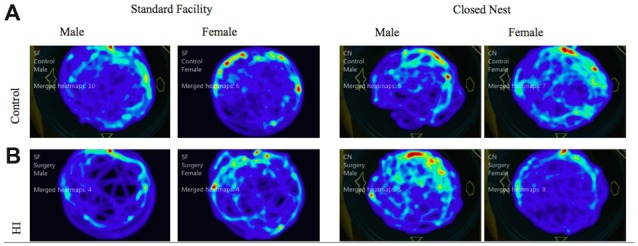
**(A,B)** Probe trial: average swim path by surgery condition and housing for males and females. The following heat maps mark the average swim path during the probe trial. The markings represent aggregate data from CN and SF housing conditions for each sex according to surgical condition: **(A)** Control, **(B)** HI Injury. The maps illustrate that HI SF animals had significantly longer swim paths than either HI CN, Control SF, or Control CN animals (*F*_(1)_ = 7.115, *p* < 0.01). For swim speed velocity, there was no difference between surgical conditions, but there was a significant effect of housing condition, with SF females demonstrating a faster swim speed than CN females (*F*_(1)_ = 7.05, *p* < 0.01).

Probe trials are conducted without a platform after VP training. These trials serve as control conditions to determine swimming speed and distance traveled as measures of locomotor ability, separate from memory function. Male animals displayed no significant differences between surgical condition or housing condition in either distance traveled or velocity of movement. Female animals however, displayed a significant interaction effect of housing condition and surgical condition, with HI SF animals demonstrating significantly longer swim paths than either HI CN, Control SF, or Control CN animals (*F*_(1)_ = 7.115, *p* < 0.01). For swim speed velocity, there was no difference between surgical conditions, but there was a significant effect of housing condition, with SF females demonstrating a faster swim speed than CN females (*F*_(1)_ = 7.05, *p* < 0.01; Figure 6). Male and female HI animals in both housing conditions demonstrated comparable swimming speed velocity and distance traveled in relation to intact animals, indicating that differences in latencies can be attributed to differential learning acquisition, search strategy, working memory and visuospatial memory ability; rather than differences in motor ability due to injury. We also have preliminary data that indicate HI injured pups show sex differences in a rope suspension task with HI-injured females reared in CN holding on to the rope longer (unpublished data). See Figure 6 for representations of aggregate swim paths.

## Expression of DAPI, and Neurotrophins BDNF and GDNF

**Figure 7:**
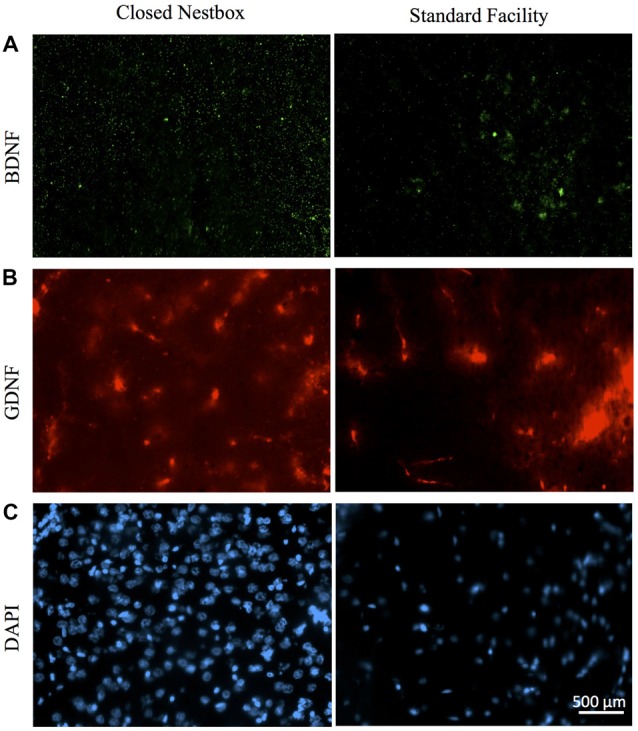
Immunoflourescent staining for neurotrophic factors and DAPI vary by surgical and housing conditions in males. **(A)** Ipsilateral hippocampal CA3 staining for BDNF in HI-injured males housed in CN (left panel) and SF (right panel). **(B)** GDNF-positive cells in males that suffered HI insult and were reared in CN (left panel) and SF (right panel). **(C)** DAPI-stained cells of male rats following HI insult and CN (left panel) and SF (right panel) pre-weaning environments. Scale bar in bottom right image = 500 μm, 20× magnification for all images.

In males, HI injury resulted in significantly lower average BDNF levels in both the hippocampus (*F*_(1,14)_ = 14.672, *p* < 0.01; Figure 7) and striatum (*F*_(1,12)_ = 40.053, *p* < 0.001; Figure 7) in comparison to control males, regardless of housing condition. We observed partial eta squared of 0.488 and 0.769, with 0.873 and 1.0 power, respectively. CN rearing increased average BDNF counts in the striatum for male subjects compared to SF males (*F*_(1,12)_ = 7.232, *p* < 0.05); here, we observed partial eta squared of 0.376, with 0.695 power. There was no interaction effect between housing condition and surgical condition on BDNF counts in males.

**Figure 8:**
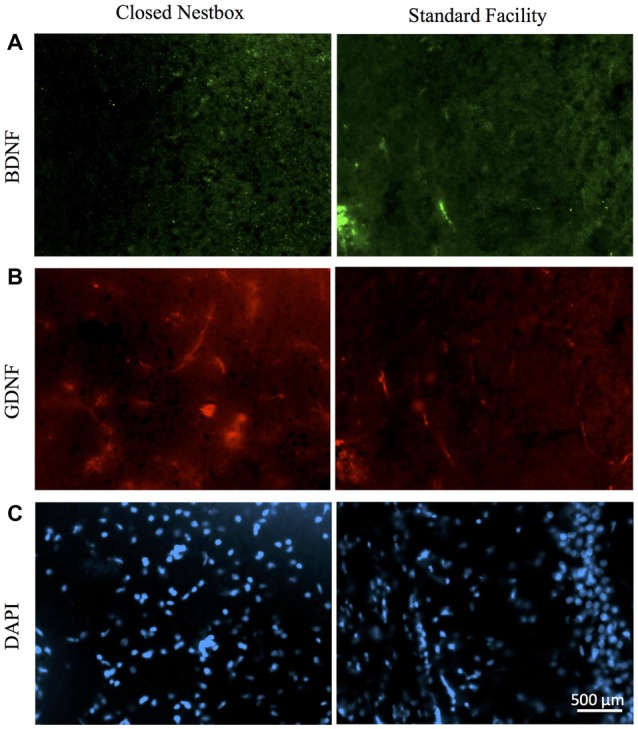
HI injury differentially affects neurotrophic factors BDNF, GDNF and DAPI in the hippocampus of male rats reared in CN and SF conditions. **(A)** BDNF-positive staining in CA3 region of hippocampi of male rats after HI injury and CN (left panel) and SF (right panel) rearing. **(B)** Anti-GDNF positive cells in the hippocampus of HI-injured male rats from CN (left panel) and SF (right panel) housing. **(C)** Staining of DAPI cells in HI males from CN (left panel) and SF (right panel) environments. Scale bar = 500 μm, 20× magnification for all images.

Females with HI did not display a lower hippocampal BDNF levels than control females (*F*_(1,13)_ = 0.068, *p* > 0.05, *NS*), however, there was a trend towards higher striatal BDNF counts for control females (*F*_(1,13)_ = 4.315, *p* = 0.058). Rearing in the CN condition resulted in higher hippocampal BDNF compared to SF females (*F*_(1,13)_ = 4.883, *p* < 0.05). There was no significant interaction effect of surgical condition and housing condition (*F*_(1,13)_ = 3.588, *p* > 0.05, *NS*). However, there was an observable trend for HI females in the CN condition to exhibit hippocampal BDNF levels similar to that of control females. HI females in the SF conditions possessed lower BDNF hippocampal counts than all other groups (Figure 8).

**Figure 9:**
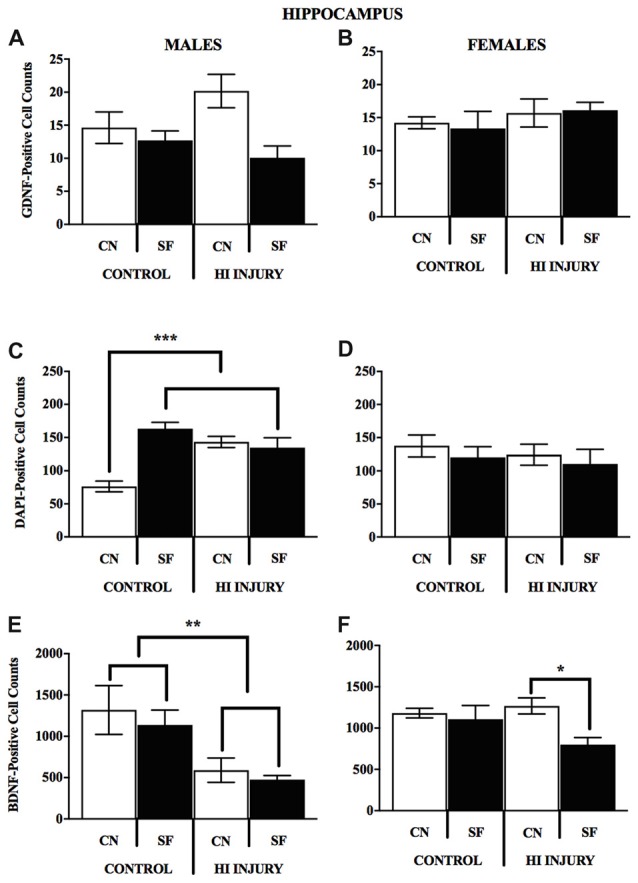
**(A–F)** Immunopositive cell counts for GDNF, DAPI and BDNF in hippocampus by surgery and housing conditions for both sexes. Bar graphs show average ±SEM counts for **(A–B)** GDNF-positive cells, **(C–D)** DAPI-positive cells and **(E–F)** BDNF-positive cells in the hippocampus. No significant main effects or interactions were observed for GDNF. For DAPI, male control rats reared in CN had fewer immunopositve cells relative to all other groups, ***p < 0.001. HI injury males reared in SF had fewer cells relative to Control males reared in the same condition, *p < 0.05, **p < 0.01.

The DNA-binding stain, DAPI (4′,6-diamidino-2-phenylindole) was used in order to both verify fluorescent staining but also to evaluate cell populations within each brain ROI. Interaction effects of surgical condition and housing condition indicated that the number of hippocampal cells present were significantly greater in control animals reared in SF conditions verses all other groups (*F*_(1,14)_ = 18.938, *p* < 0.01). Control males reared in CN conditions possessed lower levels of DAPI-stained cell bodies in the hippocampus as compared to all other groups, including all HI males. No significant differences were observed for males in striatal DAPI levels according to surgical condition (*F*_(1,14)_ = 2.650, *p* > 0.05, *NS*) or housing condition (*F*_(1,14)_ = 0.327, *p* < 0.05; Figure 9).

**Figure 10:**
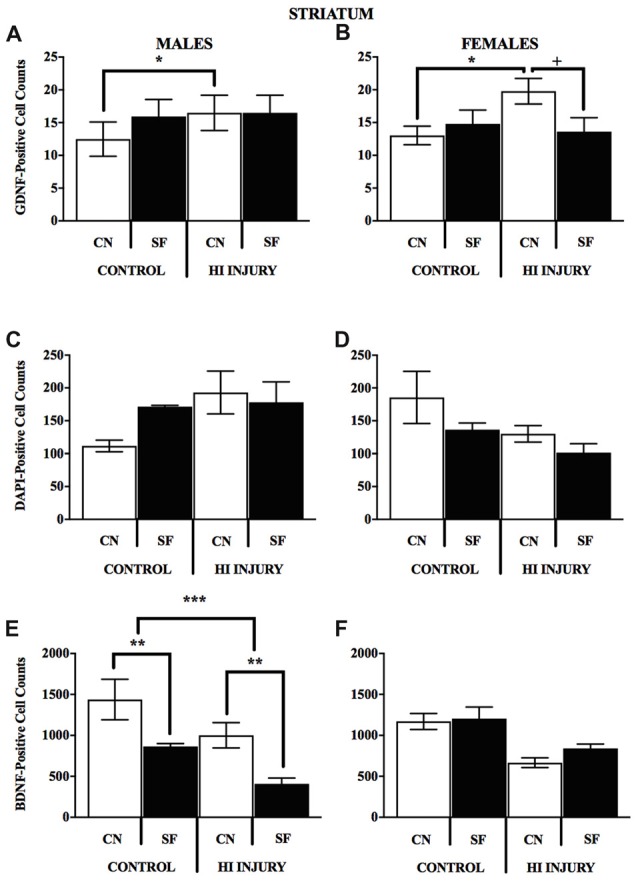
**(A–F)** Positively stained cells for BDNF GDNF, DAPI in the striatum by surgery condition and rearing in males and females. **(A-B)** In both males and females, CN rearing increased GDNF counts following HI injury, *p < 0.05, relative to Control CN; ^+^*p* < 0.05, compared to HI injury SF rearing. **(C-D)** No differences were observed for DAPI staining for males or females for any of the surgical or housing conditions. **(E)** CN rearing improved BDNF stained cells in the striatum of males, regardless of injury (control and HI); **p < 0.01, CN vs. SF, same injury group. The overall number for BDNF-positive cells was greater in the control males relative to HI injured males, ***p < 0.001.

For female animals, two-way ANOVA revealed a significant difference between HI and control females (*F*_(1,12)_ = 5.211, *p* < 0.05), with control females exhibiting significantly greater counts of DAPI-stained cells in the striatum than HI females (Figure 10). No differences in hippocampal DAPI-stained cells based on surgical condition (*F*_(1,13)_ = 1.011, *p* > 0.05, *NS*) or housing condition (*F*_(1,13)_ = 0.291, *p* = 0.599). There was also no effect of housing condition (*F*_(1,13)_ = 3.345, *p* = 0.090) on DAPI levels in the striatum of females.

Analysis of GDNF immunoreactive cells revealed a significant interaction effect between surgical condition and housing condition (*F*_(1,14)_ = 5.743, *p* < 0.05) indicating that CN improved the abundance of GDNF in the striatum of both control males and HI males (Figure 10). Although no significant differences were found between the factors of surgical and housing conditions on hippocampal GDNF, housing condition trended towards significance (*F*_(1,14)_ = 4.337, *p* = 0.056), with CN reared HI males showing the greatest levels of GDNF intensity compared to all other groups.

For females, there was a significant interaction effect between surgical condition and housing condition on GDNF counts in the striatum (*F*_(1,13)_ = 5.777, *p* < 0.05), with HI-CN females showing the highest counts of all groups. No significant differences were observed in regard to hippocampal measurement of GDNF for females or for males by housing or surgical condition, although a trend towards significance was observed in males by housing condition (*F*_(1,14)_ = 4.337, *p* = 0.056).

## Discussion

### Overview of Findings

The present study was designed to assess the potential buffering effects of enrichment in the early pre-weaning environment on the negative neurological and cognitive effects of term neonatal HI injury. Dams were given a CN environment that we posited would provide early environmental enrichment for dams and pups and might promote mCTB, as measured by pup weight gain. Indeed, even though HI injury lowered pre-weaning weights for females, CN rearing protected against weight loss during adolescence following HI insult in males and females. The closed nest environment lead to significant changes in neurotrophin levels after HI injury in females and males suggestive of neuroprotection, namely higher levels of GDNF in the striatum in HI injured animals. The observed trend for HI-CN females to have near-normal levels of hippocampal BDNF may suggest even greater neuroprotection from early environmental enrichment for females.

### Learning and Memory

Neonatal HI injury is known to result in significant white matter damage (Jansen and Low, 1996) and cognitive impairments including learning and memory deficits (Huang et al., 2009). MWM is a standardized tool for assessing visuo-spatial learning and memory in rodents (Morris, 1984; Vorhees and Williams, 2006); we performed MWM using 3 days of VP training and acquisition followed by 5 days of HP testing and a probe trial (PND 35–43) in which the platform is removed. This developmental period maps onto school-age when many survivors of neonatal HIE demonstrate deficits in learning and memory (Lindström et al., 2006).

The results of the current study indicate that all animals were able to acquire new learning during the 3-day training period regardless of injury or housing condition, however, control female animals learned the location of the platform more quickly and demonstrated shorter latencies than HI females during training, regardless of housing condition. During the testing phase, when a HP was located in a fixed position for each testing day, all animals regardless of injury, and housing condition, were able to learn the location of the HP, as indicated by decreased latencies across testing days (Hill et al., 2011). This suggests that all animals demonstrated some degree of intact long-term memory and adaptive search patterns, however, there were significant differences between injury and housing groups in the speed with which they were able to remember and locate the HP. Only female animals with HI injury demonstrated a significant functional benefit from CN housing, showing comparable latencies to those of control females in both housing conditions, and SF HI females demonstrating significant impairment in comparison. Males with HI injury demonstrated significantly longer search times, indicating poorer long-term memory and search strategies than intact males, regardless of housing condition.

Previous studies have found that, in addition to being more vulnerable to neonatal HI (Hill and Fitch, 2012), males are less responsive to some treatments for neonatal HI injury than are females (Pereira et al., 2008; Fan et al., 2011; Nie et al., 2016). Research by Pereira et al. (2008) showed a selective benefit of environmental enrichment on visuospatial memory for HI injured female animals, without having an effect on males. We have unpublished data indicating improve rope suspension performance for HI injured females but not males reared in CN (unpublished data; Mason et al., 2016). There are several mechanisms for sex differences in HI injury and recovery that have been proposed, including sex-dependent cell-death pathways (Zhu et al., 2006), protective effects of estrogen (Gerstner et al., 2009), and structural neurological differences due to early testosterone exposure (Hill et al., 2011).

Other labs have demonstrated that adolescent and adult environmental enrichment leads to improved performance in MWM for mice and rats without injury (Hullinger et al., 2015; Garthe et al., 2016). Interestingly, there were no independent effects of housing condition on water maze performance for control animals. Some research has indicated that environmental interventions may be specifically beneficial for injured or neurologically compromised animals (Pereira et al., 2008; Ravenelle et al., 2014). It may be the case that interventions targeting early nesting environment are specifically helpful for functional repair after injury and less helpful for memory performance in healthy, normally developing animals. This may be particularly true for animals exposed to neonatal HI, due to the vulnerability of hippocampal tissue (Jansen and Low, 1996; McAuliffe et al., 2006).

### Neurotrophic Factors

BDNF and GDNF likely contribute to neuroprotective effects following HI injury in neonatal rats (Abe, 2000; Jin et al., 2003) and novel treatments can be assessed through changes in these two factors (Miyazaki et al., 2001; Griesbach et al., 2004), as well as through overall number of cell bodies present (Levison et al., 2001). The neuroprotective effects of BDNF are mediated at least in part by ERK1/2 secondary signaling; this signaling blocks the activation of caspases necessary for apoptotic mechanisms following HI damage and ensuing necrotic stress (Han and Holtzman, 2000; Jones and Bergeron, 2004). Furthermore, GDNF aids in the survival of brain tissue via inflammatory proteins activated during neonatal HI, such as interleukin-6 and caspase-3 (Miyazaki et al., 2001; Kilic et al., 2003).

Overall, average BDNF level was higher in intact animals, and lower in HI animals, as has been reported (Pereira et al., 2008; Chavez-Valdez et al., 2014). CN conditions *reversed* the overall reduction in hippocampal BDNF levels after HI injury for both males and females. HI-CN conditions resulted in non-significant differences BDNF levels in females in striatal tissue. By contrast, HI CN levels were significantly higher than HI SF and control levels were greater than HI injury levels. It is likely that endogenous hormones contribute to this dimorphism in some respect since BDNF levels in intact female rats fluctuate depending on estrous cycle (Franklin and Perrot-Sinal, 2006), and with higher levels of estradiol, female rats have increased recall, general memory and spatial navigation over males (Luine and Frankfurt, 2013).

We quantified the average number of cell bodies using DAPI immunofluorescence. It was surprising that HI insult did not have a significant effect on the number of DAPI-tagged cells in the striatum of study subjects. However, we posit that the lack of specificity in this fluorescent DAPI staining—which produced coloration of all activated, double-stranded DNA-containing nuclei rather than neurons alone—could be one possible explanation for this (Levison et al., 2001). Interestingly, we did find DAPI counts in CN control males were lower than all other groups, implying reduced brain structure following environmental enrichment (van Praag et al., 2000).

No notable distinctions were observed for hippocampal GDNF staining in either males or females. However, striatal GDNF counts were significantly affected by CN rearing conditions for both females and males with HI. In females, HI-CN animals had more striatal GDNF positive cells over HI-SF and control CN animals. In a rodent model of Parkinson’s disease, viral vector transfer of BDNF and GDNF into nigrostriatal neurons was no more potent than GDNF alone (Sun et al., 2005), implying that current striatal changes in GDNF alone may underlie MWM sex differences.

Early environmental enrichment improved search patterns in the probe trial following MWM testing, regardless of injury. Despite observing similarities in swimming speed and latency across control and HI conditions, CN conditions appeared impactful for rodent navigation in the MWM. It is unlikely that induced differences between groups are due to physical movement and/or exercise related to the testing protocol. Past research has indicated that it may take 28–31 days to persistently upregulate endogenous BDNF through physical activity in the MWM (Adlard et al., 2004; Griesbach et al., 2004). While physical stimulation may indeed induce transient changes in neurotrophins from baseline (Huang et al., 2006; Ferris et al., 2007), animals in our current study were sacrificed more than 2 weeks following the conclusion of behavioral testing.

### Conclusions From Cognitive and Neurotrophic Outcomes

The neuroprotective benefit of a CN environment following HI injury was partly sex-specific. For females exposed to HI, those reared in CN demonstrated cognitive abilities similar to those of non-injured females, in contrast to HI females reared in SF housing that demonstrated significant functional impairment and diminished weight gain. There was a trend for HI females to demonstrate a similar beneficial effect of CN on neurotrophic factors, which may depend on levels of estradiol (Luine and Frankfurt, 2013). In contrast, males with HI injury showed no significant cognitive benefit from CN. However, both males and females in the HI-CN condition did demonstrate higher levels of GDNF in the striatum, indicating that CN may have afforded some neuroprotection to HI males. HI injury most prominently affects cortical matter and hippocampal tissue (Busl and Greer, 2010), and males are more likely to incur severe immediate damage to these regions than females (Liu et al., 2007; Hill and Fitch, 2012; Smith et al., 2014). In terms of recovery period, females are thought to have “depressed” metabolic function in comparison to males (Morken et al., 2014). It is plausible that CN rearing, while theoretically protective from environmental stress in the laboratory setting, was not intensive enough to sufficiently rescue the damage induced by HI for males.

The difference between male and female levels of these neurotrophins could be directly related to the availability of the nest itself from birth. Reduced nesting environment leads to “fragmented,” and stress-provoking maternal behavior in rat dams towards their offspring (Ivy et al., 2008). Adult laboratory rats do not spontaneously build nests (Van Loo and Baumans, 2004), and it has been suggested that researcher-provided materials for nesting may give rats a degree of control over their laboratory cage surroundings (as reviewed by Simpson and Kelly, 2011). Further, a small opaque enclosure similar to our CN can decrease environmental stress (Würbel, 2001). An early study by Manser et al. (1998) showed that rats will preferentially occupy an opaque nesting box with a roof and surrounding walls and a smaller entrance when offered multiple types of nesting, a design style similar to the CN provided here. Adult female rats have been hypothesized to have more complex interactions with their nesting environments as a result of hormones, and so it was expected the pregnant dams would adapt to the CN (Pietropaolo et al., 2004). Since rat dams demonstrate preferential licking and grooming for male pups, regardless of strain (Moore et al., 1997; McGowan et al., 2011), it is possible that a low-stress nursing environment provided for more equal distribution of maternal care for both sexes, thereby improving care specifically for female pups.

One future direction could include additional enrichment after weaning through the traditional social sensorimotor stimulation in housing animals with peers, toys and physical objects as it may promote further neuroplasticity through experience (Johnston et al., 2009). This may diminish apoptotic changes close to injury (Bondi et al., 2014) that we could investigate with earlier sacrifice days, and, therefore, create additive effects to the benefits presented here. In clinical populations, most infants with HIE are provided with standard-of-care treatment, therapeutic hypothermia (Eicher et al., 2005), if they can be treated within 6 h of life. Environmental enrichment may provide adjunctive benefits in combination with hypothermia. In future studies, examining the combination of treatment approaches could provide further insight into clinical applications.

## Author Contributions

LR conceived the original pilot study and worked with SD to develop a full experiment. BM supported the expansion of the work and contributed novel insights in developing the experiment, particularly molecular targets. LR and BM completed surgeries and behavioral testing with support from EA, CC and NW. LR, BM, EA, CC, NW and SD completed the terminal procedures including animal sacrifice, brain extraction and tissue preparation for immunohistochemical analyses. BM completed all immunohistochemical work. SD, LR, BM, EA, CC and NW contributed to writing the Methods and entered data for analyses. BM and LR completed statistical analyses and wrote the Results. LR and BM both contributed equally to all aspects of the manuscript writing with support from SD.

## Conflict of Interest Statement

The authors declare that the research was conducted in the absence of any commercial or financial relationships that could be construed as a potential conflict of interest. The reviewer GL and handling Editor declared their shared affiliation at the time of the review.

## References

[B1] Abe K. (2000). Therapeutic potential of neurotrophic factors and neural stem cells against ischemic brain injury. J. Cereb. Blood Flow Metab. 20, 1393–1408. 10.1097/00004647-200010000-00001 11043902

[B2] Adlard P. A. Perreau V. M. Engesser-Cesar C. Cotman C. W. (2004). The timecourse of induction of brain-derived neurotrophic factor mRNA and protein in the rat hippocampus following voluntary exercise. Neurosci. Lett. 363, 43–48. 10.1016/s0304-3940(04)00365-9 15157993

[B3] Allen S. J. Watson J. J. Shoemark D. K. Barua N. U. Patel N. K. (2013). GDNF, NGF and BDNF as therapeutic options for neurodegeneration. Pharmacol. Ther. 138, 155–175. 10.1016/j.pharmthera.2013.01.004 23348013

[B4] Almli C. R. Levy T. J. Han B. H. Shah A. R. Gidday J. M. Holtzman D. M. (2000). BDNF protects against spatial memory deficits following neonatal hypoxia-ischemia. Exp. Neurol. 166, 99–114. 10.1006/exnr.2000.7492 11031087

[B5] Arteni N. S. Salgueiro J. Torres I. Achaval M. Netto C. A. (2003). Neonatal cerebral hypoxia-ischemia causes lateralized memory impairments in the adult rat. Brain Res. 973, 171–178. 10.1016/s0006-8993(03)02436-3 12738060

[B6] Azzarelli B. Caldemeyer K. S. Phillips J. P. DeMyer W. E. (1996). Hypoxic-ischemic encephalopathy in areas of primary myelination: a neuroimaging and PET study. Pediatr. Neurol. 14, 108–116. 10.1016/0887-8994(96)00010-0 8703222

[B8] Bakshi A. Shimizu S. Keck C. A. Cho S. LeBold D. G. Morales D.. (2006). Neural progenitor cells engineered to secrete GDNF show enhanced survival, neuronal differentiation and improve cognitive function following traumatic brain injury. Eur. J. Neurosci. 23, 2119–2134. 10.1111/j.1460-9568.2006.04743.x 16630059

[B9] Balduini W. De Angelis V. Mazzoni E. Cimino M. (2000). Long-lasting behavioral alterations following a hypoxic/ischemic brain injury in neonatal rats. Brain Res. 859, 318–325. 10.1016/s0006-8993(00)01997-1 10719080

[B10] Berman A. K. Lott R. B. Donaldson S. T. (2014). Periodic maternal deprivation may modulate offspring anxiety-like behavior through mechanisms involving neuroplasticity in the amygdala. Brain Res. Bull. 101, 7–11. 10.1016/j.brainresbull.2013.12.005 24334024 PMC3939038

[B11] Björkman S. T. Miller S. M. Rose S. E. Burke C. Colditz P. B. (2010). Seizures are associated with brain injury severity in a neonatal model of hypoxia ischemia. Neuroscience 166, 157–167. 10.1016/j.neuroscience.2009.11.067 20006975

[B12] Bona E. Johansson B. B. Hagberg H. (1997). Sensorimotor function and neuropathology five to six weeks after hypoxia-ischemia in seven-day-old rats. Pediatr. Res. 42, 678–683. 10.1203/00006450-199711000-00021 9357943

[B104] Bondi C. O. Klitsch K. C. Leary J. B. Kline A. E. (2014). Environmental enrichment as a viable neurorehabilitation strategy for experimental traumatic brain injury. J. Neurotrauma 31, 873–888. 10.1089/neu.2014.3328 24555571 PMC4012629

[B13] Bredy T. Grant R. Champagne D. Meaney M. (2003). Maternal care influences neuronal survival in the hippocampus of the rat. Eur. J. Neurosci. 18, 2903–2909. 10.1111/j.1460-9568.2003.02965.x 14656341

[B14] Busl K. M. Greer D. M. (2010). Hypoxic-ischemic brain injury: pathophysiology, neuropathology and mechanisms. NeuroRehabilitation 26, 5–13. 10.3233/NRE-2010-0531 20130351

[B100] Cai J. Kang Z. Liu W. W. Luo X. Qiang S. Zhang J. H. (2008). Hydrogen therapy reduces apoptosis in neonatal hypoxia-ischemia rat model. Neurosci. Lett. 441, 167–172. 10.1016/j.neulet.2008.05.077 18603371

[B15] Caldji C. Tannenbaum B. Sharma S. Francis D. Plotsky P. M. Meaney M. J. (1998). Maternal care during infancy regulates the development of neural systems mediating the expression of fearfulness in the rat. Proc. Natl. Acad. Sci. U S A 95, 5335–5340. 10.1073/pnas.95.9.5335 9560276 PMC20261

[B16] Cameron Y. L. Merazzi D. Mortola J. P. (2000). Variability of the breathing pattern in newborn rats: effects of ambient temperature in normoxia or hypoxia. Pediatr. Res. 47, 813–818. 10.1203/00006450-200006000-00022 10832743

[B17] Champagne F. A. Francis D. D. Mar A. Meaney M. J. (2003). Variations in maternal care in the rat as a mediating influence for the effects of environment on development. Physiol. Behav. 79, 359–371. 10.1016/s0031-9384(03)00149-5 12954431

[B18] Chavez-Valdez R. Martin L. J. Razdan S. Gauda E. B. Northington F. J. (2014). Sexual dimorphism in BDNF signaling after neonatal hypoxia-ischemia and treatment with necrostatin-1. Neuroscience 260, 106–119. 10.1016/j.neuroscience.2013.12.023 24361177 PMC3950408

[B19] Chen A. I. Xiong L. J. Tong Y. U. Mao M. (2013). The neuroprotective roles of BDNF in hypoxic ischemic brain injury. Biomed. Rep. 1, 167–176. 10.3892/br.2012.48 24648914 PMC3956206

[B20] Chiaretti A. Piastra M. Polidori G. Di Rocco C. Caresta E. Antonelli A.. (2003). Correlation between neurotrophic factor expression and outcome of children with severe traumatic brain injury. Intensive Care Med. 29, 1329–1338. 10.1007/s00134-003-1852-6 12845427

[B21] Chou I. C. Trakht T. Signori C. Smith J. Felt B. T. Vazquez D. M.. (2001). Behavioral/environmental intervention improves learning after cerebral hypoxia-ischemia in rats. Stroke 32, 2192–2197. 10.1161/hs0901.095656 11546916

[B22] de Paula S. Vitola A. S. Greggio S. de Paula D. Mello P. B. Lubianca J. M.. (2009). Hemispheric brain injury and behavioral deficits induced by severe neonatal hypoxia-ischemia in rats are not attenuated by intravenous administration of human umbilical cord blood cells. Pediatr. Res. 65, 631–635. 10.1203/pdr.0b013e31819ed5c8 19430381

[B23] Demers E. J. McPherson R. J. Juul S. E. (2005). Erythropoietin protects dopaminergic neurons and improves neurobehavioral outcomes in juvenile rats after neonatal hypoxia-ischemia. Pediatr. Res. 58, 297–301. 10.1203/01.pdr.0000169971.64558.5a 16055937

[B24] McGowan P. O. Suderman M. Sasaki A. Huang T. C. Hallett M. Meaney M. J.. (2011). Broad epigenetic signature of maternal care in the brain of adult rats. PLoS One 6:e14739. 10.1371/journal.pone.0014739 21386994 PMC3046141

[B25] Eicher D. J. Wagner C. L. Katikaneni L. P. Hulsey T. C. Bass W. T. Kaufman D. A.. (2005). Moderate hypothermia in neonatal encephalopathy: efficacy outcomes. Pediatr. Neurol. 32, 11–17. 10.1016/j.pediatrneurol.2004.06.014 15607598

[B26] Fan X. Heijnen C. J. van der Kooij M. A. Groenendaal F. van Bel F. (2011). Beneficial effect of erythropoietin on sensorimotor function and white matter after hypoxia-ischemia in neonatal mice. Pediatr. Res. 69, 56–61. 10.1203/PDR.0b013e3181fcbef3 20856165

[B28] Ferris L. T. Williams J. S. Shen C. L. (2007). The effect of acute exercise on serum brain-derived neurotrophic factor levels and cognitive function. Med. Sci. Sports Exerc. 39, 728–734. 10.1249/mss.0b013e31802f04c7 17414812

[B29] Franklin T. B. Perrot-Sinal T. S. (2006). Sex and ovarian steroids modulate brain-derived neurotrophic factor (BDNF) protein levels in rat hippocampus under stressful and non-stressful conditions. Psychoneuroendocrinology 31, 38–48. 10.1016/j.psyneuen.2005.05.008 15996825

[B30] Garthe A. Roeder I. Kempermann G. (2016). Mice in an enriched environment learn more flexibly because of adult hippocampal neurogenesis. Hippocampus 26, 261–271. 10.1002/hipo.22520 26311488 PMC5049654

[B31] Gerstner B. Lee J. DeSilva T. M. Jensen F. E. Volpe J. J. Rosenberg P. A. (2009). 17β-estradiol protects against hypoxic/ischemic white matter damage in the neonatal rat brain. J. Neurosci. Res. 87, 2078–2086. 10.1002/jnr.22023 19224575 PMC2770176

[B32] Griesbach G. S. Hovda D. A. Molteni R. Wu A. Gomez-Pinilla F. (2004). Voluntary exercise following traumatic brain injury: brain-derived neurotrophic factor upregulation and recovery of function. Neuroscience 125, 129–139. 10.1016/j.neuroscience.2004.01.030 15051152

[B34] Han B. H. Holtzman D. M. (2000). BDNF protects the neonatal brain from hypoxic-ischemic injury *in vivo* via the ERK pathway. J. Neurosci. 20, 5775–5781. 10.1523/jneurosci.20-15-05775.2000 10908618 PMC6772561

[B35] Hill C. A. Fitch R. H. (2012). Sex differences in mechanisms and outcome of neonatal hypoxia-ischemia in rodent models: implications for sex-specific neuroprotection in clinical neonatal practice. Neurol. Res. Int. 2012:867531. 10.1155/2012/867531 22474588 PMC3306914

[B36] Hill C. A. Threlkeld S. W. Fitch R. H. (2011). Reprint of “Early testosterone modulated sex differences in behavioral outcome following neonatal hypoxia ischemia in rats”. Int. J. Dev. Neurosci 29, 621–628. 10.1016/j.ijdevneu.2011.07.009 21802505 PMC3960833

[B37] Huang A. M. Jen C. J. Chen H. F. Yu L. Kuo Y. M. Chen H. I. (2006). Compulsive exercise acutely upregulates rat hippocampal brain-derived neurotrophic factor. J. Neural Transm. 113, 803–811. 10.1007/s00702-005-0359-4 16252072

[B38] Huang Z. Liu J. Cheung P. Y. Chen C. (2009). Long-term cognitive impairment and myelination deficiency in a rat model of perinatal hypoxic-ischemic brain injury. Brain Res. 1301, 100–109. 10.1016/j.brainres.2009.09.006 19747899

[B39] Hullinger R. O’Riordan K. Burger C. (2015). Environmental enrichment improves learning and memory and long-term potentiation in young adult rats through a mechanism requiring mGluR5 signaling and sustained activation of p70s6k. Neurobiol. Learn Mem. 125, 126–134. 10.1016/j.nlm.2015.08.006 26341144 PMC4938427

[B40] Ikeda T. Mishima K. Yoshikawa T. Iwasaki K. Fujiwara M. Xia Y. X.. (2001). Selective and long-term learning impairment following neonatal hypoxic-ischemic brain insult in rats. Behav. Brain Res. 118, 17–25. 10.1016/s0166-4328(00)00287-4 11163630

[B41] Ivy A. S. Brunson K. L. Sandman C. Baram T. Z. (2008). Dysfunctional nurturing behavior in rat dams with limited access to nesting material: a clinically relevant model for early-life stress. Neuroscience 154, 1132–1142. 10.1016/j.neuroscience.2008.04.019 18501521 PMC2517119

[B42] Jansen E. M. Low W. C. (1996). Long-term effects of neonatal ischemic-hypoxic brain injury on sensorimotor and locomotor tasks in rats. Behav. Brain Res. 78, 189–194. 10.1016/0166-4328(95)00248-0 8864051

[B43] Jin G. Omori N. Nagano I. Manabe Y. Shoji M. Abe K. (2003). Protection against ischemic brain damage by GDNF affecting cell survival and death signals. Neurol. Res. 25, 249–253. 10.1179/016164103101201454 12739232

[B44] Johnston M. V. Ishida A. Ishida W. N. Matsushita H. B. Nishimura A. Tsuji M. (2009). Plasticity and injury in the developing brain. Brain Dev. 31, 1–10. 10.1016/j.braindev.2008.03.014 18490122 PMC2660856

[B101] Jones N. M. Bergeron M. (2004). Hypoxia-induced ischemic tolerance in neonatal rat brain involves enhanced ERK1/2 signaling. J. Neurochem. 89, 157–167. 10.1111/j.1471-4159.2004.02324.x 15030400

[B45] Kilic U. Kilic E. Dietz G. P. Bähr M. (2003). Intravenous TAT-GDNF is protective after focal cerebral ischemia in mice. Stroke 34, 1304–1310. 10.1161/01.str.0000066869.45310.50 12677018

[B46] Kiprianova I. Freiman T. M. Desiderato S. Schwab S. Galmbacher R. Gillardon F.. (1999). Brain-derived neurotrophic factor prevents neuronal death and glial activation after global ischemia in the rat. J. Neurosci. Res. 56, 21–27. 10.1002/(SICI)1097-4547(19990401)56:1<21::AID-JNR3>3.0.CO;2-Q 10213471

[B47] Koo J. W. Park C. H. Choi S. H. Kim N. J. Kim H. S. Choe J. C.. (2003). The postnatal environment can counteract prenatal effects on cognitive ability, cell proliferation and synaptic protein expression. FASEB J. 17, 1556–1558. 10.1096/fj.02-1032fje 12824278

[B49] Laviola G. Hannan A. J. Macrì S. Solinas M. Jaber M. (2008). Effects of enriched environment on animal models of neurodegenerative diseases and psychiatric disorders. Neurobiol. Dis. 31, 159–168. 10.1016/j.nbd.2008.05.001 18585920

[B50] Levison S. W. Rothstein R. P. Romanko M. J. Snyder M. J. Meyers R. L. Vannucci S. J. (2001). Hypoxia/ischemia depletes the rat perinatal subventricular zone of oligodendrocyte progenitors and neural stem cells. Dev. Neurosci. 23, 234–247. 10.1159/000046149 11598326

[B51] Lin L. F. Doherty D. H. Lile J. D. Bektesh S. Collins F. (1993). GDNF: a glial cell line-derived neurotrophic factor for midbrain dopaminergic neurons. Science 260, 1130–1132. 10.1126/science.8493557 8493557

[B52] Lindström K. Lagerros P. Gillberg C. Fernell E. (2006). Teenage outcome after being born at term with moderate neonatal encephalopathy. Pediatr. Neurol. 35, 268–274. 10.1016/j.pediatrneurol.2006.05.003 16996401

[B53] Liu M. Hurn P. D. Roselli C. E. Alkayed N. J. (2007). Role of P450 aromatase in sex-specific astrocytic cell death. J. Cereb. Blood Flow Metab. 27, 135–141. 10.1038/sj.jcbfm.9600331 16736049

[B54] Lou H. C. (1996). Etiology and pathogenesis of Attention Deficit Hyperactivity Disorder (ADHD): significance of prematurity and perinatal hypoxic haemodynamic encephalopathy. Acta Paediatr. 85, 1266–1271. 10.1111/j.1651-2227.1996.tb13909.x 8955450

[B56] Luine V. Frankfurt M. (2013). Interactions between estradiol, BDNF and dendritic spines in promoting memory. Neuroscience 239, 34–45. 10.1016/j.neuroscience.2012.10.019 23079626 PMC3597766

[B57] Manser C. E. Broom D. M. Overend P. Morris T. H. (1998). Investigations into the preferences of laboratory rats for nest-boxes and nesting materials. Lab. Anim. 32, 23–35. 10.1258/002367798780559365 9481691

[B102] Mason B. M. Romero V. Rollins L. G. Lamour S. Walton N. Londoño P. (2016). “Nesting environment impacts hypoxic-ischemic injury in rats. Program No. 791.18,” in Neuroscience Meeting Planner (San Diego, CA: Society for Neuroscience).

[B58] Martinez-Biarge M. Diez-Sebastian J. Kapellou O. Gindner D. Allsop J. M. Rutherford M. A.. (2011). Predicting motor outcome and death in term hypoxic-ischemic encephalopathy. Neurology 76, 2055–2061. 10.1212/WNL.0b013e31821f442d 21670434 PMC3111238

[B59] McAuliffe J. J. Miles L. Vorhees C. V. (2006). Adult neurological function following neonatal hypoxia-ischemia in a mouse model of the term neonate: water maze performance is dependent on separable cognitive and motor components. Brain Res. 1118, 208–221. 10.1016/j.brainres.2006.08.030 16997287

[B60] Meaney M. J. (2001). Maternal care, gene expression and the transmission of individual differences in stress reactivity across generations. Ann. Rev. Neurosci. 24, 1161–1192. 10.1146/annurev.neuro.24.1.1161 11520931

[B61] Miyazaki H. Nagashima K. Okuma Y. Nomura Y. (2001). Expression of glial cell line-derived neurotrophic factor induced by transient forebrain ischemia in rats. Brain Res. 922, 165–172. 10.1016/s0006-8993(01)03013-x 11743946

[B62] Moore C. L. Wong L. Daum M. C. Leclair O. U. (1997). Mother-infant interactions in two strains of rats: implications for dissociating mechanism and function of a maternal pattern. Dev. Psychobiol. 30, 301–312. 10.1002/(sici)1098-2302(199705)30:4<301::aid-dev4>3.0.co;2-s 9142506

[B63] Morken T. S. Brekke E. Håberg A. Widerøe M. Brubakk A. M. Sonnewald U. (2014). Altered astrocyte-neuronal interactions after hypoxia-ischemia in the neonatal brain in female and male rats. Stroke 45, 2777–2785. 10.1161/strokeaha.114.005341 25052323

[B64] Morris R. (1984). Developments of a water-maze procedure for studying spatial learning in the rat. J. Neurosci. Methods 11, 47–60. 10.1016/0165-0270(84)90007-4 6471907

[B65] Mortola J. P. Dotta A. (1992). Effects of hypoxia and ambient temperature on gaseous metabolism of newborn rats. Am. J. Physiol. 263, R267–R272. 10.1152/ajpregu.1992.263.2.r267 1510168

[B66] Mychasiuk R. Zahir S. Schmold N. Ilnytskyy S. Kovalchuk O. Gibb R. (2012). Parental enrichment and offspring development: modifications to brain, behavior and the epigenome. Behav. Brain Res. 228, 294–298. 10.1016/j.bbr.2011.11.036 22173001

[B67] Nakajima W. Ishida A. Lange M. S. Gabrielson K. L. Wilson M. A. Martin L. J.. (2000). Apoptosis has a prolonged role in the neurodegeneration after hypoxic ischemia in the newborn rat. J. Neurosci. 20, 7994–8004. 10.1523/jneurosci.20-21-07994.2000 11050120 PMC6772742

[B68] Nie X. Lowe D. W. Rollins L. G. Bentzley J. Fraser J. L. Martin R.. (2016). Sex-specific effects of N-acetylcysteine in neonatal rats treated with hypothermia after severe hypoxia-ischemia. Neurosci. Res. 108, 24–33. 10.1016/j.neures.2016.01.008 26851769 PMC4903952

[B69] Northington F. J. Zelaya M. E. O’Riordan D. P. Blomgren K. Flock D. L. Hagberg H.. (2007). Failure to complete apoptosis following neonatal hypoxia-ischemia manifests as “continuum” phenotype of cell death and occurs with multiple manifestations of mitochondrial dysfunction in rodent forebrain. Neuroscience 149, 822–833. 10.1016/j.neuroscience.2007.06.060 17961929 PMC3947608

[B70] Paxinos G. Watson C. (2004). The Rat Brain in Stereotaxic Coordinates. New York, NY: Elsevier. 10.1016/0165-0270(80)90021-7 6110810

[B71] Pereira L. O. Strapasson A. C. P. Nabinger P. M. Achaval M. Netto C. A. (2008). Early enriched housing results in partial recovery of memory deficits in female, but not in male, rats after neonatal hypoxia-ischemia. Brain Res. 1218, 257–266. 10.1016/j.brainres.2008.04.010 18514167

[B72] Perez A. Ritter S. Brotschi B. Werner H. Caflisch J. Martin E.. (2013). Long-term neurodevelopmental outcome with hypoxic-ischemic encephalopathy. J. Pediatr. 163, 454–459. 10.1016/j.jpeds.2013.02.003 23498155

[B74] Pietropaolo S. Branchi I. Chiarotti F. Alleva E. (2004). Utilisation of a physically-enriched environment by laboratory mice: age and gender differences. Appl. Ani. Behav. Sci. 88, 149–162. 10.1016/j.applanim.2004.02.015

[B75] Ravenelle R. Santolucito H. B. Byrnes E. M. Byrnes J. J. Donaldson S. T. (2014). Housing environment modulates physiological and behavioral responses to anxiogenic stimuli in trait anxiety male rats. Neuroscience 270, 76–87. 10.1016/j.neuroscience.2014.03.060 24713371 PMC4047719

[B76] Rice J. E. Vannucci R. C. Brierley J. B. (1981). The influence of immaturity on hypoxic-ischemic brain damage in the rat. Ann. Neurol. 9, 131–141. 10.1002/ana.410090206 7235629

[B77] Rojas J. J. Deniz B. F. Miguel P. M. Diaz R. do Espírito-Santo Hermel É. Achaval M.. (2013). Effects of daily environmental enrichment on behavior and dendritic spine density in hippocampus following neonatal hypoxia-ischemia in the rat. Exp. Neurol. 241, 25–33. 10.1016/j.expneurol.2012.11.026 23219882

[B78] Roth T. L. Sweatt J. D. (2011). Annual research review: epigenetic mechanisms and environmental shaping of the brain during sensitive periods of development. J. Child Psychol. Psychiatry 52, 398–408. 10.1111/j.1469-7610.2010.02282.x 20626526 PMC2965301

[B79] Silverstein F. S. Jensen F. E. (2007). Neonatal seizures. Ann. Neurol. 62, 112–120. 10.1002/ana.21167 17683087

[B80] Simpson J. Kelly J. P. (2011). The impact of environmental enrichment in laboratory rats—behavioural and neurochemical aspects. Behav. Brain Res. 222, 246–264. 10.1016/j.bbr.2011.04.002 21504762

[B81] Skaper S. D. (2012). “The neurotrophin family of neurotrophic factors: an overview,” in Methods in Molecular Biology, ed. Skaper S. D. (New York, NY: Humana Press), 1–12. 10.1007/978-1-61779-536-7_1 22367796

[B82] Smith A. L. Alexander M. Rosenkrantz T. S. Sadek M. L. Fitch R. H. (2014). Sex differences in behavioral outcome following neonatal hypoxia ischemia: insights from a clinical meta-analysis and a rodent model of induced hypoxic ischemic brain injury. Exp. Neurol. 254, 54–67. 10.1016/j.expneurol.2014.01.003 24434477

[B83] Smith D. H. Okiyama K. Thomas M. J. Claussen B. McIntosh T. K. (1991). Evaluation of memory dysfunction following experimental brain injury using the Morris water maze. J. Neurotrauma 8, 259–269. 10.1089/neu.1991.8.259 1803034

[B84] Sun M. Kong L. Wang X. Lu X. G. Gao Q. Geller A. I. (2005). Comparison of the capability of GDNF, BDNF, or both, to protect nigrostriatal neurons in a rat model of Parkinson’s disease. Brain Res. 1052, 119–129. 10.1016/j.brainres.2005.05.072 16018990 PMC2581863

[B85] Szabadfi K. Atlasz T. Horváth G. Kiss P. Hamza L. Farkas J.. (2009). Early postnatal enriched environment decreases retinal degeneration induced by monosodium glutamate treatment in rats. Brain Res. 1259, 107–112. 10.1016/j.brainres.2009.01.004 19171125

[B86] Van Loo P. L. P. Baumans V. (2004). The importance of learning young: the use of nesting material in laboratory rats. Lab. Anim. 38, 17–24. 10.1258/00236770460734353 14979984

[B87] van Praag H. Kempermann G. Gage F. H. (2000). Neural consequences of enviromental enrichment. Nat. Rev. Neurosci. 1, 191–198. 10.1038/35044558 11257907

[B88] Vannucci R. C. Connor J. R. Mauger D. T. Palmer C. Smith M. B. Towfighi J.. (1999). Rat model of perinatal hypoxic-ischemic brain damage. J. Neurosci. Res. 55, 158–163. 10.1002/(sici)1097-4547(19990115)55:2<158::aid-jnr3>3.0.co;2-1 9972818

[B89] Venable N. Fernández V. Díaz E. Pinto-Hamuy T. (1989). Effects of preweaning environmental enrichment on basilar dendrites of pyramidal neurons in occipital cortex: a Golgi study. Brain Res. 49, 140–144. 10.1016/0165-3806(89)90068-0 2791261

[B90] Volpe J. J. (2009). Brain injury in premature infants: a complex amalgam of destructive and developmental disturbances. Lancet Neurol. 8, 110–124. 10.1016/s1474-4422(08)70294-1 19081519 PMC2707149

[B91] Vorhees C. V. Williams M. T. (2006). Morris water maze: procedures for assessing spatial and related forms of learning and memory. Nat. Protoc. 1, 848–858. 10.1038/nprot.2006.116 17406317 PMC2895266

[B92] Wang W. Redecker C. Bidmon H. J. Witte O. W. (2004). Delayed neuronal death and damage of GDNF family receptors in CA1 following focal cerebral ischemia. Brain Res. 1023, 92–101. 10.1016/j.brainres.2004.07.034 15364023

[B94] Würbel H. (2001). Ideal homes? Housing effects on rodent brain and behaviour. Trends Neurosci. 24, 207–211. 10.1016/s0166-2236(00)01718-5 11250003

[B96] Zhao Y. D. Ou S. Cheng S. Y. Xiao Z. He W. J. Zhang J. H.. (2013). Dendritic development of hippocampal CA1 pyramidal cells in a neonatal hypoxia-ischemia injury model. J. Neurosci. Res. 91, 1165–1173. 10.1002/jnr.23247 23686818

[B97] Zhu C. Xu F. Wang X. Shibata M. Uchiyama Y. Blomgren K.. (2006). Different apoptotic mechanisms are activated in male and female brains after neonatal hypoxia-ischaemia. J. Neurochem. 96, 1016–1027. 10.1111/j.1471-4159.2005.03639.x 16412092

